# Cancer Malignancy Is Enhanced by Glyceraldehyde-Derived Advanced Glycation End-Products

**DOI:** 10.1155/2010/739852

**Published:** 2010-06-29

**Authors:** Jun-Ichi Takino, Sho-Ichi Yamagishi, Masayoshi Takeuchi

**Affiliations:** ^1^Department of Life Pharmacy, Faculty of Pharmaceutical Sciences, Hokuriku University, Ho-3 Kanagawa-machi, Kanazawa 920-1181, Japan; ^2^Department of Pathophysiology and Therapeutics of Diabetic Vascular Complications, Kurume University School of Medicine, 67 Asahimachi, Kurume 830-0011, Japan

## Abstract

The receptor for advanced glycation end-products (RAGEs) is associated with the malignancy of cancer. A recent study has suggested that glyceraldehyde-derived AGEs (Glycer-AGEs) enhanced the malignancy of melanoma cells, but glucose-derived AGEs did not. However, the effects of Glycer-AGEs on other cancer cells remain poorly understood, and the molecular mechanisms behind the above-mentioned effect have not been clarified. The present paper aimed to examine the effect of Glycer-AGEs on cultured lung cancer A549 cells. RAGE was expressed in A549 cells. Glycer-AGEs significantly attenuated cell proliferation. Furthermore, Glycer-AGEs enhanced the migration capacity of the cells by activating Rac1 *via* ROS and also increased their invasion capacity. We demonstrated that Glycer-AGEs enhanced the migration and invasion of A549 cells rather than their proliferation. These results suggest that Glycer-AGEs play a critical role in the malignancy of cancer rather than its proliferation and are potential targets for therapeutic intervention.

## 1. Introduction

 The main cause of treatment failure and death in cancer patients is metastasis—the formation of secondary tumors in organs distant from the original neoplastic cell tissue. Adjuvant therapy of proven efficacy is not currently available for cancer patients; therefore, the search for new targets of therapeutic reagents is required to prevent both proliferation and metastases. During metastasis, cancer cells activate matrix digestion and migration to allow their invasion across basement membranes [1]. It is known that the mode of invasion is one of the markers of the malignancy and prognosis of cancer [2]. The receptor for advanced glycation end-products (RAGE), a multiligand member of the immunoglobulin superfamily of cell surface molecules, interacts with distinct molecules implicated in homeostasis, development, and inflammation [3]. RAGE binding by ligands such as advanced glycation end-products (AGEs) triggers the activation of key cell signaling pathways, thereby reprogramming cellular properties. In addition, several reports have suggested that RAGE is associated with cancer malignancy [4, 5]. 

 The advanced stage of the glycation process (one of the posttranslational modifications of proteins) leads to the formation of AGEs and plays an important role in the pathogenesis of angiopathy in diabetic patients, aging, and neurodegenerative diseases [6–9]. A growing body of evidence suggests that the interaction of glyceraldehyde-derived AGEs (Glycer-AGEs), but not glucose-derived AGEs (Glc-AGEs), with RAGE elicits oxidative stress generation in numerous types of cells (vascular wall cells, mesangial cells, Schwann cells, and cortical neurons), all of which could contribute to the pathological changes seen in diabetic vascular complications of Alzheimer's disease [10–13]. We have recently found that Glycer-AGEs stimulated the growth and migration of cultured human melanoma cells and that anti-RAGE antibodies inhibited the tumor formation and lung metastasis of melanoma cell xenografts and subsequently improved survival in athymic mice [14]. However, the effects of Glycer-AGEs on other cancer cells remains poorly understood, and the molecular mechanisms behind their effects have not been clarified. 

 In the present study, we examined the effects of Glycer-AGEs on cultured human lung adenocarcinoma A549 cells and showed that Glycer-AGEs enhanced their malignancy rather than their proliferation.

## 2. Materials and Methods

### 2.1. Chemicals

All chemicals were commercial samples of high purity and were used as supplied. N-acetyl-L-cysteine (NAC) was purchased from Sigma-Aldrich (St. Louis, MO, USA).

### 2.2. Preparation of Glyceraldehyde-Derived AGEs (Glycer-AGEs)

Glycer-AGEs-BSA was prepared as described previously in [15]. Briefly, 25 mg/ml of BSA (A0281, Sigma-Aldrich) were incubated at 37°C for 7 days under sterile conditions with 0.1 M glyceraldehyde (GA; Nakalai Tesque, Kyoto, Japan) and 5 mM diethylenetriamine-pentaacetic acid (Dojindo, Kumamoto, Japan) in 0.2 M phosphate buffer (pH 7.4). The modified albumin was then purified by PD-10 column (GE Healthcare, Buckinghamshire, England) chromatography and dialysis against phosphate-buffered saline (PBS). Control unglycated BSA was incubated under the same conditions except for the absence of glyceraldehyde as a negative control. Protein concentrations were determined with the DC protein assay reagent (Bio-Rad, Richmond, CA, USA) using BSA as a standard. The preparations were tested for endotoxin using the Pyrotell-T test (Seikagaku Bio-business, Tokyo, Japan), but no endotoxin was detected.

### 2.3. Cell Cultures

Human lung adenocarcinoma A549 and hepatocellular carcinoma Hep3B cells were grown in Dulbecco's modified Eagle's medium (DMEM; Sigma-Aldrich) supplemented with 10% fetal bovine serum (FBS; Equitech-Bio, Kerrville, TX, USA) under standard cell culture conditions (humidified atmosphere, 5% CO_2_, 37°C). RAGE vector-, which were kindly provided by Dr. Yamagishi, and its mock vector-transfected Hep3B cells were maintained in 10% FBS/DMEM in the presence of 700 *μ*g/mL G418 (Roche, Mannheim, Germany).

### 2.4. Preparation of Cell Lysate and Western Blot Analysis

The cells were washed with ice-cold Ca^2+^ and Mg^2+^ free PBS (PBS (-)) and subjected to lysis buffer (1% TritonX-100/Nonidet P-40, 10 mM sodium fluoride, 1 mM sodium orthovanadate, 5 mM sodium pyrophosphate, 2 mM EGTA, 5 mM EDTA, and 1× protease inhibitor cocktail (complete, Mini; Roche)). Subsequently, the cell lysates were incubated on ice for 5 min and centrifuged at 10,000 *g* for 10 min at 4°C. Their protein concentrations were then measured using the Bradford assay (Bio-Rad). Cell lysates (30 *μ*g of proteins/lane) dissolved in SDS sample buffer (62.5 mM Tris-HCl (pH 6.8), 2% SDS, 10% glycerol, and 0.01% bromophenol blue) containing 5% 2-mercaptoethanol (2-ME) were boiled for 3 min at 95°C, separated by SDS-PAGE, and then electrotransferred onto PVDF membranes (Millipore, Billerica, MA, USA). Biotinylated markers (Cell Signaling, Beverly, MA, USA) were used as molecular weight markers. The membranes were blocked for 1 h using 5% skimmed milk in PBS containing 0.05% polyoxyethylene sorbitan monolaurate (PBS-T). After being washed twice with PBS-T, the membranes were incubated with goat anti-RAGE antibody (N-16) or mouse anti-*β*-actin antibody (Santa Cruz, Santa Cruz, CA, USA) for 1.5 h. Subsequently, the membranes were washed twice with PBS-T and incubated with anti-goat IgG antibody (Santa Cruz) or anti-mouse Ig's antibody (Biosource, Camarillo, CA, USA) and anti-biotin antibody (Cell Signaling) for 1 h. After being washed a further two times with PBS-T, the immunoreactive proteins were detected with ECL Plus Western Blotting Detection Reagents (GE Healthcare) using a luminescent image analyzer (LAS-1000UVmini; Fujifilm, Tokyo, Japan). The density of the bands was analyzed using a Multi Gauge version 3.0 (Fujifilm).

### 2.5. Cell Viability Assay

The cells (7×10^3^ cells/cm^2^) were seeded in various plates or culture dishes (BD Biosciences, Franklin Lakes, NJ, USA) and incubated for 24 h. The control unglycated BSA and Glycer-AGEs treatments were carried out in 0.1% FBS/DMEM for 48 and 72 h, and cell viability was determined by the WST-1 assay. After removing the medium, 100 *μ*L/well of 10% FBS/DMEM and 10 *μ*L/well of WST-1 solution (5 mM WST-1, 0.2 mM 1-methoxy-5-methylphenazinium, and 20 mM HEPES buffer (pH 7.4)) (Dojindo) were added, and the cells were incubated for 2 h. Absorbance was measured at 450 nm and 650 nm using a microplate reader (Labsystems Multiskan Ascent, Model No. 354; Thermo Fisher Scientific, Kanagawa, Japan), and the net difference (A_450_ – A_650_) was used to express cell viability.

### 2.6. Migration and Invasion Assay

The migration and invasion capacity were evaluated in 24-well transwell chambers and BioCoat Matrigel invasion chambers (BD Biosciences), respectively. In both assays, the upper and lower culture compartments were separated by polyethylene terephthalate filters with a pore size of 8 *μ*m. Ten percent FBS was used as a chemoattractant in the lower chamber compartments. Before starting the invasion assay, the Matrigel matrix of the chambers was reconstituted by adding serum-free DMEM for 2 h. In the migration assay, 1 × 10^4^ cells in serum-free DMEM with control unglycated BSA or Glycer-AGEs (100 *μ*g/ml) were added into each of the upper chambers for 20 h. In the invasion assay, 2 × 10^4^ cells in serum-free DMEM with control unglycated BSA or Glycer-AGEs were added to each of the upper chambers for 48 h. Then, the nonmigrating or noninvading cells on the upper side of the chamber membranes were removed using cotton swabs. The cells that migrated to or invaded the opposite side of the chamber were counted.

### 2.7. Rac1 Activity Assay

The active form of Rac1 was detected with the EZ-Detect Rac1 Activation Kit (Pierce, Rockford, IL, USA). This assay uses the ability of a glutathione S-transferase (GST) fusion protein corresponding to the p21-binding domain (PBD) of human p21 activated protein kinase 1 (Pak1) to specifically bind to the active GTP-bound and not the inactive GDP-bound forms of Rac1 and Cdc42. Cell lysates were incubated with GST-Pak1-PBD and SwellGel Immobilized Glutathione Discs for 1 h at 4°C, leaving an aliquot for measuring the levels of total Rac1. The bound proteins were eluted in 2× SDS sample buffer containing 5% 2-ME for 5 min at 95°C and characterized by Western blotting using monoclonal anti-Rac1 antibody. Rac1 activity was determined by densitometric quantification of the pulled-down Rac1-GTP level and normalizing it to the level of total Rac1 detected in the sample lysates.

### 2.8. Real-Time Reverse Transcription-PCR (Real-Time RT-PCR) Analysis

Total RNA was isolated from the cells with ISOGEN (Nippon Gene, Tokyo, Japan), and 50 ng of RNA were reverse transcribed into cDNA with PrimeScript RT reagent Kit (Takara, Shiga, Japan) using a GeneAmp PCR System 9700 (Perkin-Elmer Applied Biosystems, Foster City, CA, USA). Real-time PCR was performed with SYBR Premix Ex Taq using a Smart Cycler II System (Takara). The reaction mixture (25 *μ*L) contained 1× SYBR Premix Ex Taq, 0.2 *μ*M PCR forward primers, 0.2 *μ*M PCR reverse primers, and 10 ng of cDNA as a template. The primers used were as follows: TGF-*β*: 5′- GCG TGC TAA TGG TGG AAA CC -3′ and 5′- CGG AGC TCT GAT GTG TTG AAG A -3′, MMP-2: 5′-AGT CTG AAG AGC GTG AAG-3′ and 5′- CCA GGT AGG AGT GAG AAT G -3′, and *β*-actin: 5′- TCC ACC TCC AGC AGA TGT GG -3′and 5′- GCA TTT GCG GTG GAC GAT -3′. All processes were performed according to the manufacturer's instructions. The expression levels of target genes were calculated using a relative quantification method. *β*-Actin was used as an endogenous control gene in order to normalize target gene expression values.

### 2.9. Matrix Metalloproteinase-2 (MMP-2) Activity Assay

The cells were seeded and incubated for 24 h. The control unglycated BSA and Glycer-AGEs treatments were carried out in serum-free DMEM for 48 h. The conditioned medium was collected and used with a MMP-2 Biotrak activity assay system (GE Healthcare). All processes were performed according to the manufacturer's instructions. The results have been corrected for differences in cell number.

### 2.10. Statistical Analysis

All experiments were performed in duplicate and repeated at least two to three times, with each experiment yielding essentially identical results. Data are expressed as the mean ± standard deviation (SD). The significance of differences between group means was determined by one-way analysis of variance. *P* values less than .05 were considered statistically significantly.

## 3. Results

### 3.1. RAGE Expression in A549 Cells

**Figure 1:**
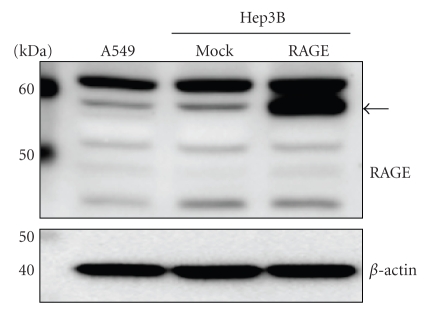
RAGE expression by Western blot analysis. Cell lysates (30 *μ*g of proteins/lane) were loaded onto a 10% polyacrylamide gel. Size markers (kDa) are shown on the left. Equal protein loading was estimated using anti-*β*-actin antibody. The arrow indicates full-length RAGE.

To investigate whether RAGE proteins are present in human lung adenocarcinoma A549 cells, we carried out Western blot analysis using anti-RAGE antibody (N-16). RAGE proteins of different molecular weights were detected in A549 cells (Figure 1). In full length RAGE cDNA-transfected human hepatocellular carcinoma Hep3B cells, the major band (57 kDa) (indicated by an arrow in Figure 1) represents the full-length RAGE protein. Likewise, the full-length RAGE protein was also detected in A549 and mock transfected Hep3B cells. No bands were detected in a neutralization experiment using blocking peptide (*data not shown*) so the other bands may represent deglycosylated RAGE proteins.

### 3.2. Effect of Glycer-AGEs on the Cell Viability of A549 Cells

**Figure 2:**
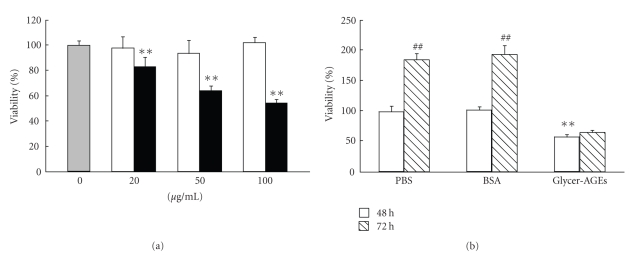
Cell viability was determined using the WST-1 assay. (a) Cells were incubated with control unglycated BSA or Glycer-AGEs for 48 h. The *shaded*, *open*, and *filled bars* represent the results for cells treated with PBS, control unglycated BSA, and Glycer-AGEs, respectively. (b) Cells were incubated with control unglycated BSA or Glycer-AGEs (100 *μ*g/ml) for 48 and 72 h. Data are shown as the mean ± SD (*n * = 6) **P < .01 versus PBS, ^##^ *P* < .01 versus 48 h.

We examined the effect of Glycer-AGEs on the cell viability of A549 cells. Cell viability was determined after 48 h incubation with control unglycated BSA or Glycer-AGEs. Twenty, 50, and 100 *μ*g/mL of Glycer-AGEs significantly decreased cell viability to 83, 64, and 54%, respectively, (Figure 2(a)). In contrast, control unglycated BSA had no effect. Furthermore, Glycer-AGEs significantly attenuated cell proliferation (Figure 2(b)); however, no cell death was induced by Glycer-AGEs according to cell death detection ELISA (*data not shown*).

### 3.3. Effects of Glycer-AGEs on Cell Migration and Invasion

**Figure 3:**
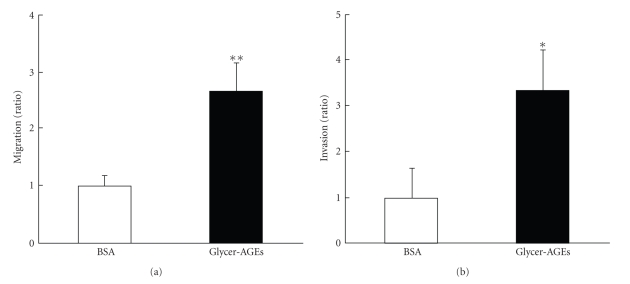
The migration and invasion capacities of A549 cells were evaluated in transwell chambers and Matrigel invasion chambers, respectively. The cells were incubated with control unglycated BSA or Glycer-AGEs for 20 h (a) or 48 h (b). The nonmigrating or non-invading cells remaining above the chamber membrane were removed with cotton swabs. The cells that migrated to or invaded the opposite side of the chamber membrane were counted. (a) Migration assay. (b) Invasion assay. Data are shown as the mean ± SD (*n* = 3) *P < .05, **P < .01 versus control unglycated BSA.

We examined the effect of Glycer-AGEs on cell migration and invasion. In the migration assay, the Glycer-AGEs-treated cells showed significantly (2.7 times higher) greater migration than the control unglycated BSA-treated cells (Figure 3(a)), and in the invasion assay, the Glycer-AGEs-treated cells showed significantly (3.3 times higher) greater invasion than the control unglycated BSA-treated cells (Figure 3(b)). When Glycer-AGEs were added to the lower chamber, there was no effect on the results of the invasion assay (*data not shown*). These results suggest that Glycer-AGEs-RAGE interaction is necessary for cell migration and invasion.

### 3.4. Glycer-AGEs Enhanced Rac1 Activity

**Figure 4:**
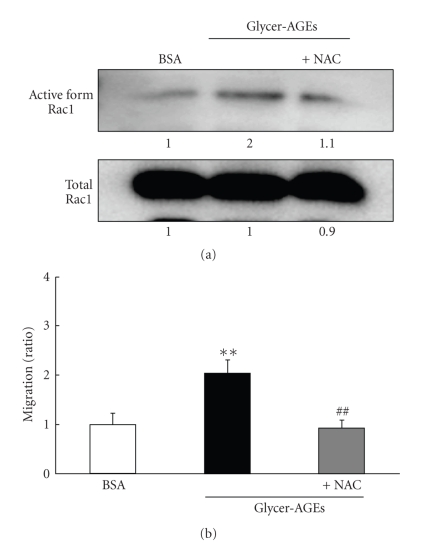
Cells were pre-incubated with or without 1 mM NAC for 30 min and then additionally incubated with control unglycated BSA or Glycer-AGEs for 1 h (a) or 20 h (b). (a) The active forms of Rac1 were precipitated with GST-Pak1-PBD and immobilized glutathione discs and analyzed by Western blotting using monoclonal anti-Rac1 antibody. At the same time, the total Rac1 in the sample lysates was detected, and the intensity of the band was quantified. (b) Migration assay. Data are shown as the mean ±  SD (*n* = 3) **P < .01 versus control unglycated BSA, ^##^ *P* < .01 versus Glycer-AGEs.

Cell migration is associated with actin reorganization, which triggers changes in cell morphology. Rho family small GTPases are widely accepted to be key regulators of actin reorganization and motility [16]. Interestingly, RAGE signaling has been shown to result in the activation of Rac1 [17–20], and the activation of RAGE by AGEs has been shown to induce the generation of reactive oxygen species (ROS) [21]. We used the Rac1 activation kit to assess activity levels. The Glycer-AGEs-induced Rac1 activity was about 2 times higher than that of the control unglycated BSA, and it was suppressed by pretreatment with N-acetyl-L-cysteine (NAC), an ROS scavenger (Figure 4(a)). Likewise, the Glycer-AGEs-enhanced migration capacity was also significantly suppressed by pre-treatment with NAC (Figure 4(b)). Thus, Glycer-AGEs-RAGE interaction enhances migration capacity by activating Rac1 *via* ROS generation.

### 3.5. Glycer-AGEs Enhanced the MMP-2 Activity

**Figure 5:**
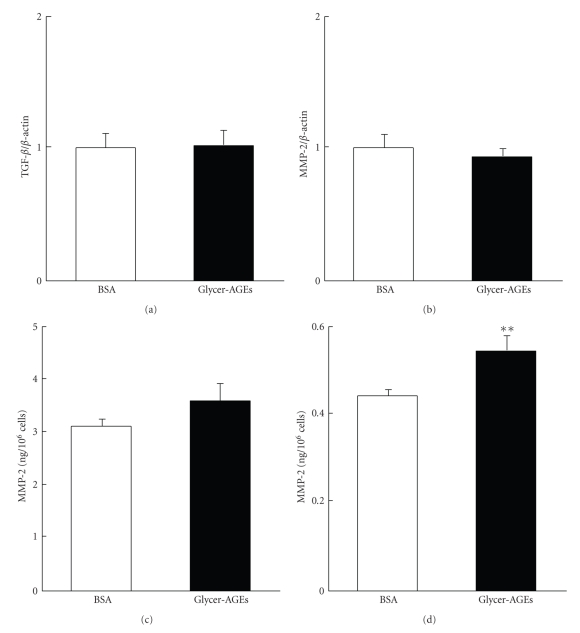
(a) and (b) Cells were incubated with control unglycated BSA or Glycer-AGEs for 24 h. The levels of TGF-*β* (a) and MMP-2 (b) mRNA expression were analyzed using the real-time RT-PCR method, and the result was normalized to the *β*-actin mRNA level. (c) and (d) Cells were incubated with control unglycated BSA or Glycer-AGEs for 48 h. The conditioned medium was collected, and the activities of pro-MMP-2 (c) and MMP-2 (the activated form) were measured (d). Data are shown as the mean ± SD (*n* = 3) **P < .01 versus control unglycated BSA.

Members of the matrix metalloproteinase (MMP) family are widely accepted to be key regulators of tumor invasion [22–24]. In particular, MMP-2 and -9, which are called gelatinases, are key enzymes for degrading type IV collagen and are thought to play critical roles in tumor invasion and metastasis [25]. We examined the expression of transforming growth factor-*β* (TGF-*β*) and MMP-2 mRNA and assessed MMP-2 activity with the MMP-2 Biotrak activity assay system. Neither the mRNA expression of TGF-*β* or MMP-2 was significantly increased by the addition of Glycer-AGEs at 24 h (Figures 5(a) and 5(b)). Pro-MMP-2 activity was also not significantly altered by the addition of Glycer-AGEs at 48 h (Figure 5(c)). However, Glycer-AGEs only increased MMP-2 (the activated form) activity to 120% (Figure 5(d)).

## 4. Discussion

 RAGE, which is a multiligand receptor, affects diseases such as cancer and diabetes through its ligands. Several reports have suggested that RAGE and amphoterin are closely associated with invasion and metastasis in cancer cells [26, 27]. AGEs have been implicated in the development and progression of diabetic angiopathies, including skin dermopathy. However, it is still unclear which AGEs subtypes play a pathogenetic role and which AGEs receptors mediate the effects of AGEs on cells. We have provided direct immunochemical evidence for the existence of six distinct AGEs structures within the AGEs-modified proteins and peptides that circulate in the sera of diabetic patients. Recently, we found that Glycer-AGEs perform a diverse range of biological activities on vascular wall cells, mesangial cells, Schwann cells, and cortical neurons [10–13]. We also found that Glycer-AGEs, but not glucose-derived AGEs (Glc-AGEs), significantly stimulated the growth and migration of human melanoma cells [14]. Furthermore, the tumor formation of melanoma cells xenografts in athymic mice was prevented by treatment with anti-RAGE antibody. In tumor bearing-mice, survival rates were prolonged, and spontaneous pulmonary metastases were inhibited by treatment using anti-RAGE antibody. In addition, Glycer-AGEs were present in the beds of human melanoma tumors, whereas they were hardly detected in normal skin. These results suggest that Glycer-AGEs are involved in the growth and invasion of malignant melanoma through their interactions with RAGE. Recently, we found that Glycer-AGEs have the strongest binding affinity for RAGE [28, 29] and subsequently elicit oxidative stress generation and evoke vascular inflammation, thereby implicating them in accelerated atherosclerosis in diabetes [10, 11]. Taken together, Glycer-AGEs-RAGE interactions play an important role in the progression of melanoma cells to malignancy. However, the effects of Glycer-AGEs on other cancer cells remain poorly understood, and the molecular mechanisms behind these effects have not been clarified. 

In the present study, we demonstrated that RAGE was expressed in A549 cells. RAGE includes full-length, C-truncated, and N-truncated RAGE [29]. The RAGE antibody (N-16) used in this study, which recognizes the N-terminus of RAGE, detects full-length and C-truncated RAGE. Indeed, full-length RAGE (57 kDa) was detected in A549 cells. The other bands may have represented the de-glycosylated form or C-truncated RAGE [30]. Next, we found that Glycer-AGEs attenuated cell proliferation in A549 cells. Glycer-AGEs are associated with cell cytotoxicity, but Glc-AGEs are not [21, 31, 32]. In mesangial cells, intracellular ROS produced by Glycer-AGEs were found to induce apoptotic cell death [21]. However, no A549 cell death induced by Glycer-AGEs was observed in this study. It is thought that the cytotoxicity of Glycer-AGEs depends on the sensitivity of their target cells to ROS. Furthermore, Glycer-AGEs enhanced the migration and invasion activity of the A549 cells, both of which are prominent features of cancer malignancy [2].

Glycer-AGEs induced the invasion of A549 cells across Matrigel, indicating that matrix degradation and migration mechanisms had been stimulated in these cells. At the molecular level, Glycer-AGEs-induced phenomena resemble the effects of long-term oxidative stress or TGF-*β*. Both oxidative stress and TGF-*β* are key regulators of the malignancy rather than the proliferation of cancer cells [33–35]. TGF-*β* strongly induces MMP-2 expression in A549 cells [36]; however, mRNA expression of TGF-*β* was not induced by Glycer-AGEs, and the mRNA levels of MMP-2, which is produced by TGF-*β*, did not change. In long-term oxidative stress, Mori et al. showed that intracellularly produced ROS activated Rac1 [33] and enhanced the invasion capacity of tumor cells by activating MMP [33, 37–39]. A recent report showed that sustained exposure of cells to H_2_O_2_, but not one time exposure to H_2_O_2_, increased pro-MMP-2 activation through the induction of membrane type 1-MMP (MT1-MMP) expression [38]. Glycer-AGEs-RAGE signaling may cause the sustained production of ROS. Indeed, our results also showed that Glycer-AGEs enhanced the migration capacity of A549 cells by activating Rac1 *via* ROS and increased their invasion capacity by activating MMP-2. However, Glycer-AGEs only increased MMP-2 (the activated form) activity to 120%. It is suggested that the activation of other MMP such as MMP-13 may also participate in the above-mentioned changes, and future studies are necessary to clarify this matter.

In conclusion, we demonstrated that Glycer-AGEs enhanced the migration and invasion of A549 cells rather than their proliferation. These results suggest that Glycer-AGEs play a critical role in cancer malignancy and are potential targets for therapeutic intervention.

## References

[1] Liotta LA, Kohn EC (2001). The microenvironment of the tumour-host interface. *Nature*.

[2] Fidler IJ (1973). Selection of successive tumour lines for metastasis. *Nature New Biology*.

[3] Schmidt AM, Yan SD, Yan SF, Stern DM (2001). The multiligand receptor RAGE as a progression factor amplifying immune and inflammatory responses. *Journal of Clinical Investigation*.

[4] Bhawal UK, Ozaki Y, Nishimura M (2005). Association of expression of receptor for advanced glycation end products and invasive activity of oral squamous cell carcinoma. *Oncology*.

[5] Kuniyasu H, Oue N, Wakikawa A (2002). Expression of receptors for advanced glycation end-products (RAGE) is closely associated with the invasive and metastatic activity of gastric cancer. *Journal of Pathology*.

[6] Al-Abed Y, Kapurniotu A, Bucala R (1999). Advanced glycation end products: detection and reversal. *Methods in Enzymology*.

[7] Takeuchi M, Makita Z (2001). Alternative routes for the formation of immunochemically distinct advanced glycation end-products in vivo. *Current Molecular Medicine*.

[8] Vlassara H, Palace MR (2002). Diabetes and advanced glycation endproducts. *Journal of Internal Medicine*.

[9] Takeuchi M, Kikuchi S, Sasaki N (2004). Involvement of advanced glycation end-products (AGEs) in Alzheimer’s disease. *Current Alzheimer Research*.

[10] Yamagishi S-I, Imaizumi T (2005). Diabetic vascular complications: pathophysiology, biochemical basis and potential therapeutic strategy. *Current Pharmaceutical Design*.

[11] Sato T, Iwaki M, Shimogaito N, Wu X, Yamagishi S-I, Takeuchi M (2006). TAGE (Toxic AGEs) theory in diabetic complications. *Current Molecular Medicine*.

[12] Sato T, Shimogaito N, Wu X, Kikuchi S, Yamagishi S-I, Takeuchi M (2006). Toxic advanced glycation end products (TAGE) theory in Alzheimer’s disease. *American Journal of Alzheimer’s Disease and Other Dementias*.

[13] Takeuchi M, Yamagishi S-I (2009). Involvement of toxic AGEs (TAGE) in the pathogenesis of diabetic vascular complications and Alzheimer’s disease. *Journal of Alzheimer’s Disease*.

[14] Abe R, Shimizu T, Sugawara H (2004). Regulation of human melanoma growth and metastasis by AGE-AGE receptor interactions. *Journal of Investigative Dermatology*.

[15] Takeuchi M, Makita Z, Bucala R, Suzuki T, Koike T, Kameda Y (2000). Immunological evidence that non-carboxymethyllysine advanced glycation end-products are produced from short chain sugars and dicarbonyl compounds in vivo. *Molecular Medicine*.

[16] Hall A (1998). Rho GTPases and the actin cytoskeleton. *Science*.

[17] Huttunen HJ, Fages C, Rauvala H (1999). Receptor for advanced glycation end products (RAGE)-mediated neurite outgrowth and activation of NF-*κ*B require the cytoplasmic domain of the receptor but different downstream signaling pathways. *Journal of Biological Chemistry*.

[18] Kuniyasu H, Chihara Y, Kondo H (2003). Differential effects between amphoterin and advanced glycation end products on colon cancer cells. *International Journal of Cancer*.

[19] Kuniyasu H, Yano S, Sasaki T, Sasahira T, Sone S, Ohmori H (2005). Colon cancer cell-derived high mobility group 1/amphoterin induces growth inhibition and apoptosis in macrophages. *American Journal of Pathology*.

[20] Yoshida T, Yamagishi S-I, Nakamura K (2006). Pigment epithelium-derived factor (PEDF) inhibits advanced glycation end product (AGE)-induced C-reactive protein expression in hepatoma cells by suppressing Rac-1 activation. *FEBS Letters*.

[21] Yamagishi S-I, Inagaki Y, Okamoto T (2002). Advanced glycation end product-induced apoptosis and overexpression of vascular endothelial growth factor and monocyte chemoattractant protein-1 in human-cultured mesangial cells. *Journal of Biological Chemistry*.

[22] Nagaset H, Woessner JF (1999). Matrix metalloproteinases. *Journal of Biological Chemistry*.

[23] Egeblad M, Werb Z (2002). New functions for the matrix metalloproteinases in cancer progression. *Nature Reviews Cancer*.

[24] Overall CM, López-Otín C (2002). Strategies for MMP inhibition in cancer: innovations for the post-trial era. *Nature Reviews Cancer*.

[25] Westermarck J, Kähäri V-M (1999). Regulation of matrix metalloproteinase expression in tumor invasion. *FASEB Journal*.

[26] Kuniyasu H, Chihara Y, Takahashi T (2003). Co-expression of receptor for advanced glycation end products and the ligand amphoterin associates closely with metastasis of colorectal cancer. *Oncology Reports*.

[27] Takada M, Koizumi T, Toyama H, Suzuki Y, Kuroda Y (2001). Differential expression of RAGE in human pancreatic carcinoma cells. *Hepato-Gastroenterology*.

[28] Yamamoto Y, Yonekura H, Watanabe T (2007). Short-chain aldehyde-derived ligands for RAGE and their actions on endothelial cells. *Diabetes Research and Clinical Practice*.

[29] Yonekura H, Yamamoto Y, Sakurai S (2003). Novel splice variants of the receptor for advanced glycation end-products expressed in human vascular endothelial cells and pericytes, and their putative roles in diabetes-induced vascular injury. *Biochemical Journal*.

[30] Osawa M, Yamamoto Y, Munesue S (2007). De-N-glycosylation or G82S mutation of RAGE sensitizes its interaction with advanced glycation endproducts. *Biochimica et Biophysica Acta*.

[31] Takeuchi M, Bucala R, Suzuki T (2000). Neurotoxicity of advanced glycation end-products for cultured cortical neurons. *Journal of Neuropathology and Experimental Neurology*.

[32] Yamagishi S-I, Amano S, Inagaki Y (2002). Advanced glycation end products-induced apoptosis and overexpression of vascular endothelial growth factor in bovine retinal pericytes. *Biochemical and Biophysical Research Communications*.

[33] Mori K, Shibanuma M, Nose K (2004). Invasive potential induced under long-term oxidative stress in mammary epithelial cells. *Cancer Research*.

[34] Kaminska B, Wesolowska A, Danilkiewicz M (2005). TGF beta signalling and its role in tumour pathogenesis. *Acta Biochimica Polonica*.

[35] Jakowlew SB (2006). Transforming growth factor-*β* in cancer and metastasis. *Cancer and Metastasis Reviews*.

[36] Kasai H, Allen JT, Mason RM, Kamimura T, Zhang Z (2005). TGF-*β*1 induces human alveolar epithelial to mesenchymal cell transition (EMT). *Respiratory Research*.

[37] Siwik DA, Pagano PJ, Colucci WS (2001). Oxidative stress regulates collagen synthesis and matrix metalloproteinase activity in cardiac fibroblasts. *American Journal of Physiology*.

[38] Yoon SO, Park SJ, Yoon SY, Yun CH, Chung AS (2002). Sustained production of H(2)O(2) activates pro-matrix metalloproteinase-2 through receptor tyrosine kinases/phosphatidylinositol 3-kinase/NF-kappa B pathway. *Journal of Biological Chemistry*.

[39] Zhang HJ, Zhao W, Venkataraman S (2002). Activation of matrix metalloproteinase-2 by overexpression of manganese superoxide dismutase in human breast cancer MCF-7 cells involves reactive oxygen species. *Journal of Biological Chemistry*.

